# dCROX and ROX Indices Predict Clinical Outcomes in Patients with COVID-19 Pneumonia Treated with High-Flow Nasal Cannula Oxygen Therapy

**DOI:** 10.1155/2024/8880259

**Published:** 2024-02-28

**Authors:** Pitchayapa Ruchiwit, Kanpisut Pongtongkam, Narongkorn Saiphoklang

**Affiliations:** Division of Pulmonary and Critical Care Medicine, Department of Internal Medicine, Faculty of Medicine, Thammasat University, Pathum Thani, Thailand

## Abstract

**Background:**

High-flow nasal cannula (HFNC) therapy is a common respiratory support in patients with COVID-19 pneumonia. Predictive tools for the evaluation of successful weaning from HFNC therapy for COVID-19 pneumonia have been limited. This study aimed to develop a new predictor for weaning success from HFNC treatment in patients with COVID-19 pneumonia.

**Methods:**

We conducted a retrospective cohort study at Thammasat University Hospital, Thailand. Patients with COVID-19 pneumonia requiring HFNC therapy from April 2020 to September 2021 were included. The ROX index was defined as the ratio of oxygen saturation (SpO_2_)/fraction of inspired oxygen (FiO_2_) to respiratory rate. The CROX index was defined as the ratio of C-reactive protein (CRP) to the ROX index. dCROX was defined as the difference in CROX index between 24 hours and 72 hours. Weaning success was defined as the ability to sustain spontaneous breathing after separation from HFNC without any invasive or noninvasive ventilatory support for ≥48 hours or death.

**Results:**

A total of 106 patients (49.1% male) were included. The mean age was 62.1 ± 16.2 years. Baseline SpO_2_/FiO_2_ was 276.1 ± 124.8. The rate of HFNC weaning success within 14 days was 61.3%. The best cutoff value of the dCROX index to predict HFNC weaning success was 3.15 with 66.2% sensitivity, 70.7% specificity, and an area under the ROC curve (AUC) of 0.71 (95% CI: 0.59–0.81, *p* < 0.001). The best cutoff value of the ROX index was 9.13, with 75.4% sensitivity, 78.0% specificity, and an AUC of 0.79 (95% CI: 0.69–0.88, *p* < 0.001).

**Conclusions:**

ROX index has the highest accuracy for predicting successful weaning from HFNC in patients with COVID-19 pneumonia. dCROX index is the alternative tool for this setting. However, a larger prospective cohort study is needed to verify these indices for determining separation from HFNC therapy. This trial is registered with TCTR20221107004.

## 1. Introduction

Coronavirus disease 2019 (COVID-19) is an infectious disease caused by coronavirus 2019 (SARS-CoV-2), first reported in 2019 at Wuhan in China, and has since spread worldwide [1]. The spectrum of disease varies from asymptomatic, upper respiratory symptoms to respiratory failure and death [2]. High-flow nasal cannula (HFNC) is one of the most common respiratory supports for patients with COVID-19 pneumonia to reduce the intubation and mortality rates [3]. It delivers a gas flow rate of up to 60 L/min, humidified and heated up to 37°C via a cannula, and maintains a constant fraction of inspired oxygen (FiO_2_). This system is associated with improvements in the washout of nasopharyngeal dead space and lung mucociliary clearance [4]. It improves oxygen delivery and increases the tolerance for patients requiring ventilatory support [4]. These patients need to be monitored by some parameters. The ROX index is a simple bedside calculation using three clinical variables: the oxygen saturation level (SpO_2_), the inspired oxygen fraction (FiO_2_), and the respiratory rate (RR) [4, 5]. It is one easy way to summarize a patient's degree of hypoxemic respiratory failure and a predictor for the need for intubation in post-HFNC therapy. It shows accuracy in predicting HFNC failure at 12 hours of treatment with the cutoff value <4.88 associated with intubation (hazard ratio, 0.27; 95% CI, 0.12 to 0.62, *p* = 0.002) [4]. However, this parameter can easily vary throughout the day or in different clinical situations, leading to possible biases.

Currently, several research studies [6–9] use serum C-reactive protein (CRP) to monitor the clinical outcomes of patients with COVID-19 pneumonia, along with other laboratory investigations. Serum CRP has been identified as an important marker in patients with severe COVID-19 [10]. This protein is produced by the liver and serves as an early marker of infection and inflammation [11]. It rapidly rises within 6 to 8 hours and reaches its highest peak in 48 hours from the disease onset [12]. CRP concentration, with a half‐life of 19 hours, falls when the inflammation or tissue damage is resolved; therefore, it is a useful marker for monitoring disease severity [13, 14].

There are several studies using the ROX index or CRP to predict intubation in patients with COVID-19 pneumonia, but there are limited data on these parameters for predicting weaning outcomes from HFNC. This study proposed a new predictor, dCROX, calculated from the changes in CRP level divided by the ROX index. The aim of this study was to determine dCROX and ROX indices to predict successful weaning from HFNC therapy in patients with COVID-19 pneumonia.

## 2. Materials and Methods

### 2.1. Study Design and Participants

This was a retrospective cohort study conducted at Thammasat University Hospital in Thailand. Patient data from electronic medical records between April 2020 and September 2021 were extracted. Patients aged 18 years and older who had COVID-19 pneumonia and were treated with HFNC for at least 48 hours were included. Patients with insufficient record data, end-stage disease, or palliative care were excluded. Demographics, comorbidities, smoking history, and laboratory data including serum CRP level, SpO_2_, and RR were collected. Clinical outcomes of weaning from HFNC (success or failure) at days 7 and 14 after starting HFNC therapy were recorded.

The ROX index was calculated from the ratio of SpO_2_ divided by FiO_2_ to RR (SpO_2_/FiO_2_/RR). ROX was collected at 24 and 72 hours after the first HFNC therapy. The CROX index was defined as the ratio of CRP to the ROX index. The delta-CROX (dCROX) index was defined as the difference in CROX index between 24 hours and 72 hours after the initiation of HFNC treatment.

Weaning success was defined as the ability to sustain spontaneous breathing after separation from HFNC without any invasive or noninvasive ventilatory support for ≥48 hours or death. Clinical outcomes of weaning from HFNC were recorded on days 7 and 14. The attending physicians supervised the doctors and nurses in charge of the weaning process. All team members underwent prestudy training on weaning from HFNC. They reduced the HFNC O_2_ flow to 10–20 liters per minute and then switched to a conventional O_2_ nasal cannula at 4 liters per minute, gradually tapering off the use of this cannula.

Ethical approval was obtained from the Human Research Ethics Committee of Thammasat University (Medicine), Thailand (IRB No. MTU-EC-IM-0-134/65, COA No. 214/2022), in full compliance with international guidelines such as the Declaration of Helsinki, The Belmont Report, CIOMS Guidelines, and the International Conference on Harmonisation-Good Clinical Practice (ICH-GCP). All methods were performed in accordance with these guidelines and regulations. Written informed consent was waived because this study was a retrospective study. This study followed the Standards for Reporting Diagnostic Accuracy Studies (STARD) to ensure the completeness and transparency of reporting in diagnostic accuracy studies.

### 2.2. Outcomes

The primary outcome was the best cutoff values of dCROX and ROX indices to predict weaning success in patients with COVID-19 pneumonia treated with HFNC. The secondary outcome was the prevalence of successful weaning in these patients.

### 2.3. Statistical Analysis

The study of CRP for predicting successful weaning from HFNC in patients with COVID-19 pneumonia has not been investigated. However, a previous study by Mueller et al. [14] revealed that a CRP increase after hospitalization of 13 mg/L within 48 hours indicates the need for advanced respiratory support. We hypothesize that the difference in CRP levels in our study was only half as low as that in the mentioned study. This hypothesis is based on our observations in the hospital, where we found an average change in CRP levels of 6 mg/L at 48 hours after the admission of COVID-19 pneumonia patients with acute respiratory failure. Consequently, we posit that a CRP level of 6 mg/L and a standard deviation of 15 can effectively distinguish between the weaning failure and successful weaning groups. We calculated the required sample size for a two-sample comparison of the mean difference using 80% power and a two-sided alpha of 0.05, resulting in a sample size of 99 participants.

Data were expressed as number (%) and mean ± standard deviation. The chi-squared test was used to compare categorical data between the successful weaning and the failed weaning groups. Student's *t*-test was used to compare continuous data between the two groups. The receiver operator characteristic (ROC) curve was used to determine the best cutoff values of dCROX and ROX indices to predict successful weaning from HFNC. The area under the ROC curve (AUC) was presented with the diagnostic ability to distinguish between two groups. A two-sided *p* value <0.05 was considered statistically significant. Statistical analyses were performed using SPSS version 26.0 software (IBM Corp., Armonk, NY, USA).

## 3. Results

### 3.1. Participants

**Figure 1:**
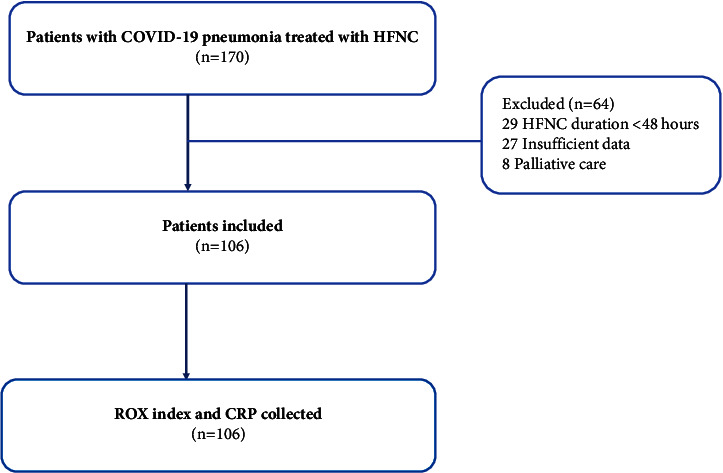
Flowchart of recruitment to the study for hospitalized patients with COVID-19 pneumonia. HFNC = high-flow nasal cannula.

A total of 170 patients with hospitalized COVID-19 pneumonia with HFNC therapy were screened, and 64 patients were excluded (Figure 1). One hundred and six patients were eligible for the final analysis. 52 (49.1%) patients were men. The mean age was 62.1 ± 16.2 years. The body mass index was 27.3 ± 5.6 kg/m^2^. Baseline SpO_2_/FiO_2_ was 276.1 ± 124.8. The most common comorbidities were hypertension (47.2%), diabetes (35.8%), and dyslipidemia (31.1%). The initial CRP concentration was 92.0 ± 63.4 mg/L (Table 1).

### 3.2. dCROX and ROX Indices

**Figure 2:**
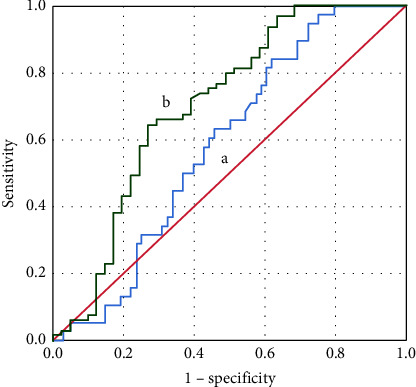
The receiver operating characteristic (ROC) plot of the dCROX index for predicting successful weaning from high-flow nasal cannula therapy within 7 days (a) and 14 days (b). The best cutoff values of the dCROX index to predict HFNC weaning success within 7 and 14 days are 3.78 and 3.15, with the area under the ROC curve of 0.59 and 0.71, respectively.

**Figure 3:**
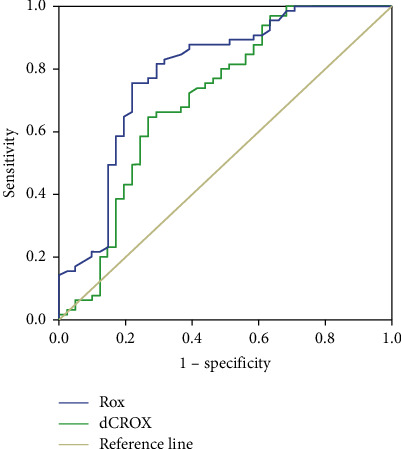
The receiver operating characteristic (ROC) plot of dCROX and ROX indices for predicting successful weaning from high-flow nasal cannula therapy within 14 days. The best cutoff values of dCROX and ROX indices to predict HFNC weaning success within 14 days are 3.15 and 9.13, with the area under the ROC curve of 0.71 and 0.79, respectively.

Patients with COVID-19 pneumonia successfully weaned off HFNC within 7 days and 14 days were 38 (35.8%) and 65 (61.3%), respectively (Table 1). The best cutoff value of the dCROX index to predict HFNC weaning success within 7 days was 3.78 with a sensitivity of 57.9%, a specificity of 57.4%, and an AUC of 0.59 (95% CI: 0.48–0.69, *p* < 0.001). The best cutoff value of the dCROX index to predict HFNC weaning success within 14 days was 3.15 with a sensitivity of 66.2%, a specificity of 70.7%, and an AUC of 0.71 (95% CI: 0.60–0.81, *p* < 0.001) (Table 2, Figure 2). The best cutoff value of the ROX index to predict HFNC weaning success within 14 days was 9.13 with a sensitivity of 75.4%, a specificity of 78.0%, and an AUC of 0.79 (95% CI: 0.69–0.88, *p* < 0.001) (Table 2, Figure 3).

## 4. Discussion

This is the first cohort study determining the addition of serum CRP to the ROX index for predicting successful weaning from HFNC treatment in hospitalized patients with COVID-19 pneumonia. Our results indicate that the ROX index had the highest sensitivity and specificity, whereas the dCROX index showed acceptable discrimination between weaning success and failure groups.

ROX values of <4.88 accurately predict the need for intubation at 12 hours due to HFNC failure (AUC, 0.74; 95% CI, 0.64 to 0.84, *p* < 0.002) [4]. In patients with successful separation from HFNC, the ROX index was higher, which was initially developed to predict the success of HFNC use with greater accuracy than any one parameter alone in severe pneumonia [5]. Our study showed that the best ROX threshold predicting successful weaning from HFNC within 14 days was 9.13. Our results are consistent with those of a retrospective observational study by Rodriguez et al. [15]. They demonstrated that a ROX index of  ≥9.2 and FiO_2_  ≤ 40% were two predictors of successful removal from HFNC at the bedside among 190 patients with acute respiratory failure treated with HFNC in the intensive care unit.

There have been many studies demonstrating a significant correlation between serum CRP levels and worsening outcomes in patients with COVID-19 pneumonia. CRP is also used to monitor the response of COVID-19 treatment. A higher CRP level is associated with poor clinical outcomes [16], whereas a decreased level of CRP is associated with the resolution of COVID-19 pneumonia [17]. A retrospective study of 298 COVID-19 patients conducted in China by Luo et al. [7] found that a CRP cutoff level >41.4 mg/L was a predictor of an adverse outcome, with an AUC of 0.896 (*p* < 0.05), 90.5% sensitivity, 77.6% specificity, 61.3% positive predictive value (PPV), and 95.4% negative predictive value (NPV). A cross-sectional study of 143 COVID-19 pneumonia patients by Wang et al. [18] found that a CRP level >64.79 mg/L was associated with a higher risk of COVID-19 progressing to a critical stage. A retrospective study by Ahnach et al. [19] showed that CRP levels at admission had good accuracy in predicting COVID-19 severity (AUC of 0.872). Our study showed that the initial CRP concentration was 91.7 ± 63.1 mg/L, which indicates a high risk of severity and poor clinical outcomes in our population. Our results found that weaning failure within 7 and 14 days from HFNC was 64.2% and 38.7%, respectively.

Our study tried to improve the accuracy of the ROX index for predicting successful weaning from HFNC by incorporating serum CRP to reduce the variability of ROX parameters, especially respiratory rates, which continued throughout the day. However, the study results did not align with our expectations, as the dCROX index failed to outperform the ROX index in predicting weaning outcomes. Our results showed that the AUCs for optimal cutoff values of dCROX and ROX for predicting HFNC weaning success within 14 days were 0.71 and 0.79, respectively. It might be explained by serum CRP, a nonspecific blood biomarker that can increase in patients with underlying conditions, such as venous thromboembolism (VTE), acute kidney injury, and critical illness [20]. These comorbid conditions might be found in our study; however, our research did not collect data on these potential confounding factors.

This study has some limitations. Firstly, the study was conducted in a single research center in Thailand; the results might not be applicable to other countries. Secondly, factors associated with CRP elevation such as VTE were not collected; therefore, high CPR levels might not reflect a certain COVID-19 severity. Lastly, parameters for ROX calculation can easily change throughout the day or in different clinical situations, leading to possible biases. The timing of parameter recording at the same time every day in patients without treatment activity can decrease the interference of study results. A large prospective study is required to validate dCROX and ROX indices for predicting weaning success in patients with COVID-19 pneumonia treated with HFNC.

## 5. Conclusions

The ROX index has the highest accuracy for predicting successful weaning from HFNC in patients with COVID-19 pneumonia. The dCROX index is the alternative tool for this setting. However, a larger prospective cohort study is needed to verify these indices for determining separation from HFNC therapy.

## Figures and Tables

**Table 1:** Baseline characteristics of hospitalized patients with COVID-19 pneumonia receiving high-flow nasal cannula treatment.

Characteristic	Data (*N* = 106)
Age (years)	62.1 ± 16.2
Male	52 (49.1)
Female	54 (50.9)
Body mass index (kg/m^2^)	27.3 ± 5.6
Comorbidity	
Hypertension	50 (47.2)
Diabetes	38 (35.8)
Dyslipidemia	33 (31.1)
Chronic kidney disease	11 (10.4)
Cardiovascular disease	5 (4.7)
Malignancy	4 (3.8)
Cerebrovascular disease	3 (2.8)
COPD	3 (2.8)
Asthma	2 (1.9)
Laboratory data	
Hemoglobin (g/dL)	12.9 ± 2.1
White blood cells count (*μ*L)	7,276.4 ± 3,478.7
Neutrophils (%)	73.5 ± 13.1
Lymphocytes (%)	17.7 ± 10.4
Platelet (*μ*L)	207,679.2 ± 93,513.9
CRP at admission (mg/L)	92.0 ± 63.4
CRP at 72 hours (mg/L)	52.0 ± 47.3
SpO_2_/FiO_2_ ratio	276.1 ± 124.8
Outcomes of weaning from HFNC	
Success within 7 days	38 (35.8)
Success within 14 days	65 (61.3)

Data shown as *n* (%) or the mean ± SD. COPD = chronic obstructive pulmonary disease, CRP = C-reactive protein, FiO_2_ = fraction of inspired oxygen, HFNC = high-flow nasal cannula, and SpO_2_ = oxygen saturation.

**Table 2:** Cutoff values of dCROX and ROX indices for predicting successful weaning from high-flow nasal cannula treatment.

Index	Cutoff value	AUC	95% CI	Sensitivity (%)	Specificity (%)	PPV (%)	NPV (%)	*p* value
dCROX (7 days^a^)	3.78	0.59	0.48–0.69	57.9	57.4	43.1	71.0	<0.001
dCROX (14 days^b^)	3.15	0.71	0.60–0.81	66.2	70.7	78.2	57.0	<0.001
ROX (14 days^b^)	9.13	0.79	0.69–0.88	75.4	78.0	84.5	66.7	<0.001

^a^Successful weaning from high-flow nasal cannula treatment within 7 days. ^b^Successful weaning from high-flow nasal cannula treatment within 14 days. AUC = area under the ROC curve, CI = confidence interval, NPV = negative predictive values, and PPV = positive predictive values.

## Data Availability

The data supporting the results of this study are available within the article.
